# Supplementary material for: Pyruvate Dehydrogenase Kinase 1 inhibition mediated oxidative phosphorylation enhancement in cartilage promotes osteoarthritis progression
Source: BMC Musculoskelet Disord. 2023 Jul 20;24:597. doi: 10.1186/s12891-023-06585-6 (PMC10357736; doi:10.1186/s12891-023-06585-6)
Supplement: Supplementary file 1 — Additional file 1. [file 12891_2023_6585_MOESM1_ESM.zip › MF.Gsea.1653627316242/GOMF_STRUCTURAL_CONSTITUENT_OF_CYTOSKELETON.html]

Details for gene set GOMF\_STRUCTURAL\_CONSTITUENT\_OF\_CYTOSKELETON[GSEA]

|  || Dataset | 55457\_collapsed\_to\_symbols.cls.cls #OA\_versus\_normal.cls.cls #OA\_versus\_normal\_repos |
| Phenotype | cls.cls#OA\_versus\_normal\_repos |
| Upregulated in class | normal |
| GeneSet | GOMF\_STRUCTURAL\_CONSTITUENT\_OF\_CYTOSKELETON |
| Enrichment Score (ES) | -0.35602674 |
| Normalized Enrichment Score (NES) | -1.5467002 |
| Nominal p-value | 0.0015822785 |
| FDR q-value | 0.15883268 |
| FWER p-Value | 1.0 |
Table: GSEA Results Summary

  

Fig 1: Enrichment plot: GOMF\_STRUCTURAL\_CONSTITUENT\_OF\_CYTOSKELETON      
 Profile of the Running ES Score & Positions of GeneSet Members on the Rank Ordered List

  

| SYMBOL | TITLE | RANK IN GENE LIST | RANK METRIC SCORE | RUNNING ES | CORE ENRICHMENT || 1 | INA | internexin neuronal intermediate filament protein alpha [Source:HGNC Symbol;Acc:HGNC:6057] | 538 | 0.645 | -0.0167 | No |
| 2 | ARPC5 | actin related protein 2/3 complex subunit 5 [Source:HGNC Symbol;Acc:HGNC:708] | 1118 | 0.492 | -0.0425 | No |
| 3 | NEFL | neurofilament light chain [Source:HGNC Symbol;Acc:HGNC:7739] | 1239 | 0.469 | -0.0335 | No |
| 4 | SORBS2 | sorbin and SH3 domain containing 2 [Source:HGNC Symbol;Acc:HGNC:24098] | 1707 | 0.397 | -0.0544 | No |
| 5 | ARPC3 | actin related protein 2/3 complex subunit 3 [Source:HGNC Symbol;Acc:HGNC:706] | 1743 | 0.393 | -0.0417 | No |
| 6 | TUBD1 | tubulin delta 1 [Source:HGNC Symbol;Acc:HGNC:16811] | 2046 | 0.356 | -0.0512 | No |
| 7 | ANK1 | ankyrin 1 [Source:HGNC Symbol;Acc:HGNC:492] | 2052 | 0.356 | -0.0376 | No |
| 8 | TUBA3E | tubulin alpha 3e [Source:HGNC Symbol;Acc:HGNC:20765] | 2169 | 0.342 | -0.0333 | No |
| 9 | ARPC1B | actin related protein 2/3 complex subunit 1B [Source:HGNC Symbol;Acc:HGNC:704] | 2184 | 0.340 | -0.0210 | No |
| 10 | KRT19 | keratin 19 [Source:HGNC Symbol;Acc:HGNC:6436] | 2258 | 0.331 | -0.0137 | No |
| 11 | ACTA1 | "actin alpha 1, skeletal muscle [Source:HGNC Symbol;Acc:HGNC:129]" | 2428 | 0.311 | -0.0147 | No |
| 12 | ADD3 | adducin 3 [Source:HGNC Symbol;Acc:HGNC:245] | 2462 | 0.307 | -0.0052 | No |
| 13 | KRT14 | keratin 14 [Source:HGNC Symbol;Acc:HGNC:6416] | 2699 | 0.279 | -0.0126 | No |
| 14 | ARPC2 | actin related protein 2/3 complex subunit 2 [Source:HGNC Symbol;Acc:HGNC:705] | 2893 | 0.261 | -0.0174 | No |
| 15 | YEATS4 | YEATS domain containing 4 [Source:HGNC Symbol;Acc:HGNC:24859] | 2933 | 0.257 | -0.0104 | No |
| 16 | TUBB | tubulin beta class I [Source:HGNC Symbol;Acc:HGNC:20778] | 3296 | 0.221 | -0.0299 | No |
| 17 | CYLC1 | cylicin 1 [Source:HGNC Symbol;Acc:HGNC:2582] | 3470 | 0.205 | -0.0354 | No |
| 18 | DES | desmin [Source:HGNC Symbol;Acc:HGNC:2770] | 3570 | 0.197 | -0.0354 | No |
| 19 | ANK3 | ankyrin 3 [Source:HGNC Symbol;Acc:HGNC:494] | 3921 | 0.163 | -0.0563 | No |
| 20 | PPL | periplakin [Source:HGNC Symbol;Acc:HGNC:9273] | 3999 | 0.156 | -0.0562 | No |
| 21 | TPM1 | tropomyosin 1 [Source:HGNC Symbol;Acc:HGNC:12010] | 4220 | 0.135 | -0.0681 | No |
| 22 | KRT16 | keratin 16 [Source:HGNC Symbol;Acc:HGNC:6423] | 4277 | 0.130 | -0.0673 | No |
| 23 | KRT20 | keratin 20 [Source:HGNC Symbol;Acc:HGNC:20412] | 4384 | 0.122 | -0.0708 | No |
| 24 | TUBG1 | tubulin gamma 1 [Source:HGNC Symbol;Acc:HGNC:12417] | 4487 | 0.114 | -0.0743 | No |
| 25 | VILL | villin like [Source:HGNC Symbol;Acc:HGNC:30906] | 4710 | 0.095 | -0.0879 | No |
| 26 | TUBGCP3 | tubulin gamma complex associated protein 3 [Source:HGNC Symbol;Acc:HGNC:18598] | 4722 | 0.094 | -0.0851 | No |
| 27 | TUBB2B | tubulin beta 2B class IIb [Source:HGNC Symbol;Acc:HGNC:30829] | 4777 | 0.089 | -0.0858 | No |
| 28 | CYLC2 | cylicin 2 [Source:HGNC Symbol;Acc:HGNC:2583] | 4926 | 0.078 | -0.0943 | No |
| 29 | TUBAL3 | tubulin alpha like 3 [Source:HGNC Symbol;Acc:HGNC:23534] | 5043 | 0.070 | -0.1006 | No |
| 30 | SPTAN1 | "spectrin alpha, non-erythrocytic 1 [Source:HGNC Symbol;Acc:HGNC:11273]" | 5131 | 0.062 | -0.1050 | No |
| 31 | TLN2 | talin 2 [Source:HGNC Symbol;Acc:HGNC:15447] | 5161 | 0.059 | -0.1050 | No |
| 32 | SPTBN2 | "spectrin beta, non-erythrocytic 2 [Source:HGNC Symbol;Acc:HGNC:11276]" | 5200 | 0.056 | -0.1057 | No |
| 33 | TUBA8 | tubulin alpha 8 [Source:HGNC Symbol;Acc:HGNC:12410] | 5208 | 0.056 | -0.1041 | No |
| 34 | SYNM | synemin [Source:HGNC Symbol;Acc:HGNC:24466] | 5300 | 0.048 | -0.1093 | No |
| 35 | ANK2 | ankyrin 2 [Source:HGNC Symbol;Acc:HGNC:493] | 5320 | 0.046 | -0.1090 | No |
| 36 | TUBG2 | tubulin gamma 2 [Source:HGNC Symbol;Acc:HGNC:12419] | 5444 | 0.037 | -0.1171 | No |
| 37 | TUBB1 | tubulin beta 1 class VI [Source:HGNC Symbol;Acc:HGNC:16257] | 5561 | 0.028 | -0.1251 | No |
| 38 | DMD | dystrophin [Source:HGNC Symbol;Acc:HGNC:2928] | 5605 | 0.023 | -0.1275 | No |
| 39 | TUBB4A | tubulin beta 4A class IVa [Source:HGNC Symbol;Acc:HGNC:20774] | 5801 | 0.008 | -0.1424 | No |
| 40 | EPB41 | erythrocyte membrane protein band 4.1 [Source:HGNC Symbol;Acc:HGNC:3377] | 5841 | 0.006 | -0.1452 | No |
| 41 | PLS1 | plastin 1 [Source:HGNC Symbol;Acc:HGNC:9090] | 5859 | 0.005 | -0.1464 | No |
| 42 | EPB41L3 | erythrocyte membrane protein band 4.1 like 3 [Source:HGNC Symbol;Acc:HGNC:3380] | 5927 | 0.000 | -0.1516 | No |
| 43 | BFSP2 | beaded filament structural protein 2 [Source:HGNC Symbol;Acc:HGNC:1041] | 5979 | -0.003 | -0.1555 | No |
| 44 | KRT2 | keratin 2 [Source:HGNC Symbol;Acc:HGNC:6439] | 6000 | -0.004 | -0.1569 | No |
| 45 | AGRN | agrin [Source:HGNC Symbol;Acc:HGNC:329] | 6155 | -0.017 | -0.1682 | No |
| 46 | KRT6A | keratin 6A [Source:HGNC Symbol;Acc:HGNC:6443] | 6628 | -0.052 | -0.2031 | No |
| 47 | KRT15 | keratin 15 [Source:HGNC Symbol;Acc:HGNC:6421] | 6643 | -0.053 | -0.2021 | No |
| 48 | BFSP1 | beaded filament structural protein 1 [Source:HGNC Symbol;Acc:HGNC:1040] | 6886 | -0.071 | -0.2182 | No |
| 49 | MSN | moesin [Source:HGNC Symbol;Acc:HGNC:7373] | 6919 | -0.074 | -0.2178 | No |
| 50 | DSP | desmoplakin [Source:HGNC Symbol;Acc:HGNC:3052] | 6931 | -0.074 | -0.2158 | No |
| 51 | KRT6B | keratin 6B [Source:HGNC Symbol;Acc:HGNC:6444] | 7008 | -0.080 | -0.2185 | No |
| 52 | ACTR3 | actin related protein 3 [Source:HGNC Symbol;Acc:HGNC:170] | 7013 | -0.081 | -0.2157 | No |
| 53 | LORICRIN | loricrin cornified envelope precursor protein [Source:HGNC Symbol;Acc:HGNC:6663] | 7036 | -0.082 | -0.2141 | No |
| 54 | KRT9 | keratin 9 [Source:HGNC Symbol;Acc:HGNC:6447] | 7055 | -0.084 | -0.2122 | No |
| 55 | HLA-DRB1 | "major histocompatibility complex, class II, DR beta 1 [Source:HGNC Symbol;Acc:HGNC:4948]" | 7087 | -0.086 | -0.2113 | No |
| 56 | SPTB | "spectrin beta, erythrocytic [Source:HGNC Symbol;Acc:HGNC:11274]" | 7127 | -0.090 | -0.2108 | No |
| 57 | EPB42 | erythrocyte membrane protein band 4.2 [Source:HGNC Symbol;Acc:HGNC:3381] | 7228 | -0.098 | -0.2147 | No |
| 58 | CD2AP | CD2 associated protein [Source:HGNC Symbol;Acc:HGNC:14258] | 7262 | -0.100 | -0.2134 | No |
| 59 | TUBB4B | tubulin beta 4B class IVb [Source:HGNC Symbol;Acc:HGNC:20771] | 7370 | -0.108 | -0.2175 | No |
| 60 | KRT31 | keratin 31 [Source:HGNC Symbol;Acc:HGNC:6448] | 7440 | -0.114 | -0.2184 | No |
| 61 | ACTR2 | actin related protein 2 [Source:HGNC Symbol;Acc:HGNC:169] | 7498 | -0.118 | -0.2182 | No |
| 62 | TUBA4A | tubulin alpha 4a [Source:HGNC Symbol;Acc:HGNC:12407] | 7668 | -0.132 | -0.2262 | No |
| 63 | ACTL6B | actin like 6B [Source:HGNC Symbol;Acc:HGNC:160] | 8002 | -0.155 | -0.2461 | No |
| 64 | EPB41L4B | erythrocyte membrane protein band 4.1 like 4B [Source:HGNC Symbol;Acc:HGNC:19818] | 8113 | -0.164 | -0.2483 | No |
| 65 | ACTL7B | actin like 7B [Source:HGNC Symbol;Acc:HGNC:162] | 8255 | -0.175 | -0.2524 | No |
| 66 | SPTA1 | "spectrin alpha, erythrocytic 1 [Source:HGNC Symbol;Acc:HGNC:11272]" | 8312 | -0.179 | -0.2498 | No |
| 67 | SPTBN1 | "spectrin beta, non-erythrocytic 1 [Source:HGNC Symbol;Acc:HGNC:11275]" | 8335 | -0.181 | -0.2444 | No |
| 68 | NEFH | neurofilament heavy chain [Source:HGNC Symbol;Acc:HGNC:7737] | 8458 | -0.190 | -0.2464 | No |
| 69 | TUBGCP4 | tubulin gamma complex associated protein 4 [Source:HGNC Symbol;Acc:HGNC:16691] | 8703 | -0.209 | -0.2572 | No |
| 70 | NEFM | neurofilament medium chain [Source:HGNC Symbol;Acc:HGNC:7734] | 8763 | -0.213 | -0.2535 | No |
| 71 | TLN1 | talin 1 [Source:HGNC Symbol;Acc:HGNC:11845] | 9131 | -0.242 | -0.2726 | No |
| 72 | SORBS3 | sorbin and SH3 domain containing 3 [Source:HGNC Symbol;Acc:HGNC:30907] | 9337 | -0.258 | -0.2785 | No |
| 73 | ARPC4 | actin related protein 2/3 complex subunit 4 [Source:HGNC Symbol;Acc:HGNC:707] | 9522 | -0.276 | -0.2820 | No |
| 74 | TUBB6 | tubulin beta 6 class V [Source:HGNC Symbol;Acc:HGNC:20776] | 10471 | -0.370 | -0.3415 | Yes |
| 75 | SPTBN4 | "spectrin beta, non-erythrocytic 4 [Source:HGNC Symbol;Acc:HGNC:14896]" | 10519 | -0.375 | -0.3304 | Yes |
| 76 | CCDC6 | coiled-coil domain containing 6 [Source:HGNC Symbol;Acc:HGNC:18782] | 10570 | -0.381 | -0.3193 | Yes |
| 77 | GFAP | glial fibrillary acidic protein [Source:HGNC Symbol;Acc:HGNC:4235] | 10757 | -0.399 | -0.3182 | Yes |
| 78 | PLEC | plectin [Source:HGNC Symbol;Acc:HGNC:9069] | 10799 | -0.405 | -0.3054 | Yes |
| 79 | TUBA3C | tubulin alpha 3c [Source:HGNC Symbol;Acc:HGNC:12408] | 10884 | -0.415 | -0.2957 | Yes |
| 80 | ACTL7A | actin like 7A [Source:HGNC Symbol;Acc:HGNC:161] | 10901 | -0.417 | -0.2806 | Yes |
| 81 | TUBB2A | tubulin beta 2A class IIa [Source:HGNC Symbol;Acc:HGNC:12412] | 10972 | -0.427 | -0.2693 | Yes |
| 82 | CROCC | "ciliary rootlet coiled-coil, rootletin [Source:HGNC Symbol;Acc:HGNC:21299]" | 11059 | -0.439 | -0.2587 | Yes |
| 83 | ADD2 | adducin 2 [Source:HGNC Symbol;Acc:HGNC:244] | 11298 | -0.471 | -0.2588 | Yes |
| 84 | CTNNA2 | catenin alpha 2 [Source:HGNC Symbol;Acc:HGNC:2510] | 11342 | -0.477 | -0.2434 | Yes |
| 85 | KRT5 | keratin 5 [Source:HGNC Symbol;Acc:HGNC:6442] | 11588 | -0.513 | -0.2424 | Yes |
| 86 | POTEKP | "POTE ankyrin domain family member K, pseudogene [Source:HGNC Symbol;Acc:HGNC:30182]" | 11812 | -0.552 | -0.2381 | Yes |
| 87 | TUBA1B | tubulin alpha 1b [Source:HGNC Symbol;Acc:HGNC:18809] | 11888 | -0.569 | -0.2216 | Yes |
| 88 | TUBA1A | tubulin alpha 1a [Source:HGNC Symbol;Acc:HGNC:20766] | 11974 | -0.589 | -0.2051 | Yes |
| 89 | TUBA1C | tubulin alpha 1c [Source:HGNC Symbol;Acc:HGNC:20768] | 12129 | -0.626 | -0.1925 | Yes |
| 90 | BICD1 | BICD cargo adaptor 1 [Source:HGNC Symbol;Acc:HGNC:1049] | 12520 | -0.757 | -0.1932 | Yes |
| 91 | ERBIN | erbb2 interacting protein [Source:HGNC Symbol;Acc:HGNC:15842] | 12535 | -0.765 | -0.1642 | Yes |
| 92 | ACTG1 | actin gamma 1 [Source:HGNC Symbol;Acc:HGNC:144] | 12613 | -0.806 | -0.1386 | Yes |
| 93 | ACTB | actin beta [Source:HGNC Symbol;Acc:HGNC:132] | 12757 | -0.920 | -0.1136 | Yes |
| 94 | VIM | vimentin [Source:HGNC Symbol;Acc:HGNC:12692] | 12777 | -0.932 | -0.0784 | Yes |
| 95 | HIP1 | huntingtin interacting protein 1 [Source:HGNC Symbol;Acc:HGNC:4913] | 12858 | -1.081 | -0.0422 | Yes |
| 96 | TUBA4B | tubulin alpha 4b [Source:HGNC Symbol;Acc:HGNC:18637] | 12878 | -1.155 | 0.0017 | Yes |
Table: GSEA details [plain text format]

  

Fig 2: GOMF\_STRUCTURAL\_CONSTITUENT\_OF\_CYTOSKELETON      
 Blue-Pink O' Gram in the Space of the Analyzed GeneSet

  

Fig 3: GOMF\_STRUCTURAL\_CONSTITUENT\_OF\_CYTOSKELETON: Random ES distribution      
 Gene set null distribution of ES for **GOMF\_STRUCTURAL\_CONSTITUENT\_OF\_CYTOSKELETON**
